# Quantitative analysis of peroxisome tracks using a Hidden Markov Model

**DOI:** 10.1038/s41598-023-46812-7

**Published:** 2023-11-11

**Authors:** Carl-Magnus Svensson, Katharina Reglinski, Wolfgang Schliebs, Ralf Erdmann, Christian Eggeling, Marc Thilo Figge

**Affiliations:** 1 Applied Systems Biology, Leibniz Institute for Natural Product Research and Infection Biology - Hans Knöll Institute, Jena, Germany; 2 Leibniz-Institute of Photonic Technologies, Jena, Germany; 3 Institute of Applied Optics and Biophysics, Friedrich-Schiller University Jena, Jena, Germany; 4 MRC Human Immunology Unit, Weatherall Institute of Molecular Medicine, University of Oxford, Oxford, UK; 5 University Hospital Jena, Jena, Germany; 6 Institute of Biochemistry and Pathobiochemistry, Systems Biochemistry, Ruhr-University Bochum, Bochum, Germany; 7 Jena Center for Soft Matter (JCSM), Jena, Germany; 8 Institute of Microbiology, Faculty of Biological Sciences, Friedrich-Schiller University Jena, Jena, Germany

**Keywords:** Protein transport, Peroxisomes, Cellular imaging, Computational models, Statistical methods

## Abstract

Diffusion and mobility are essential for cellular functions, as molecules are usually distributed throughout the cell and have to meet to interact and perform their function. This also involves the cytosolic migration of cellular organelles. However, observing such diffusion and interaction dynamics is challenging due to the high spatial and temporal resolution required and the accurate analysis of the diffusional tracks. The latter is especially important when identifying anomalous diffusion events, such as directed motions, which are often rare. Here, we investigate the migration modes of peroxisome organelles in the cytosol of living cells. Peroxisomes predominantly migrate randomly, but occasionally they bind to the cell's microtubular network and perform directed migration, which is difficult to quantify, and so far, accurate analysis of switching between these migration modes is missing. We set out to solve this limitation by experiments and analysis with high statistical accuracy. Specifically, we collect temporal diffusion tracks of thousands of individual peroxisomes in the HEK 293 cell line using two-dimensional spinning disc fluorescence microscopy at a high acquisition rate of 10 frames/s. We use a Hidden Markov Model with two hidden states to (1) automatically identify directed migration segments of the tracks and (2) quantify the migration properties for comparison between states and between different experimental conditions. Comparing different cellular conditions, we show that the knockout of the peroxisomal membrane protein *PEX14* leads to a decrease in the directed movement due to a lowered binding probability to the microtubule. However, it does not eradicate binding, highlighting further microtubule-binding mechanisms of peroxisomes than via *PEX14*. In contrast, structural changes of the microtubular network explain perceived eradication of directed movement by disassembly of microtubules by Nocodazole-treatment.

## Introduction

Accurate observation and analysis of diffusional tracks in cells are essential for understanding cellular processes, as molecules have to move to meet each other, interact and start signaling. However, accuracy is challenged by the sufficient spatial and temporal resolution of the observation technique, such as optical microscopy, and often, even more importantly, by data analysis. Directly recorded spatiotemporal diffusion tracks are usually compromised by noise and anomalous diffusion, such as local confinements or directed motions, which may be relatively rare^1–5^. This also holds for organelles in the cellular cytosol, such as endocytosed or transported vesicles or migration of peroxisomes.

Peroxisomes are ubiquitous organelles in eukaryotic cells fulfilling many metabolic functions. In humans, they are involved in the synthesis of many metabolites, such as bile acid, cholesterol and ether lipids like plasmalogens^6^. This highlights the physiological importance of peroxisomes. For example, the malfunction of plasmalogen biosynthesis is linked to severe neurological and developmental disorders^7,8^. Special properties of peroxisomes are their import of folded proteins and their migration modes throughout the cytosol^6,7,9,10^.

Two different migrations modes have been highlighted for peroxisomes in mammalian cells. A slow, random or vibrational movement can be detected for most of the peroxisomes, while a smaller fraction of peroxisomes moves in a fast, directional manner. While in the random migration mode, peroxisomes are most likely tethered to the endoplasmic reticulum (ER) or other organelles^11,12^, the directed migration is caused by guidance along microtubules using kinesin and dynein motors in mammalian cells^9,13,14^. Besides these motor proteins, the peroxisomal surface protein *PEX14* also has a role in microtubule interaction^10,15,16^. It has been reported that the directed movement of peroxisomes is impaired in *PEX14-*deficient patient cells^15^, and the loss of *PEX14* inhibits the association with the spindle pole during mitosis^17^. The interaction of *PEX14* with tubulin has been shown in pull-down assays^15^ and in in vitro studies^16,18^. The latter led to the conclusion that the cytosolic peroxisomal import protein *PEX5* and tubulin compete about binding to *PEX14*, suggesting a mechanism that links the distribution of peroxisomes to their import activity^16^.

All these findings suggest that the movement of peroxisomes is a highly regulated process that is not fully understood. However, the classification between these two types of modes depends on the experimental settings of the different studies. For example, mostly a certain speed threshold was rather arbitrarily defined to distinguish between a "fast" and a "slow" peroxisomal population^9,13,19^. Further, peroxisomes are not intrinsically "fast" or "slow" but change their migration modes over time, switching between the "slow" random migration mode and the "fast" microtubule-directed migration mode, which has so far not been investigated. Therefore, we studied this migration behavior and its dynamics with a high temporal resolution and a high statistical power, thereby tackling limitations in previous studies of the peroxisomal movement, such as (1) an insufficient temporal and spatial resolution or (2) a limited field of view that obstructed analyses of peroxisome movement and interaction with microtubules^20,21^. Even more recent studies used only a few hundred tracks to investigate migration dynamics^19,22^. We established an analysis to distinguish between the two types of peroxisomal movement using a Hidden Markov Model (HMM) on a large sample size of 628.957 tracks across three experimental conditions with a high temporal resolution of 10 frames/s. The microscopy data was obtained in two spatial dimensions, but due to the flat morphology of HEK 293 cells, the vast majority of the microtubular network was still captured in our setting.

The analysis of peroxisome movement and interaction with their environment falls into the group of single-particle track analysis. Here, we present a custom-designed HMM used to identify the directed migration of peroxisomes, allowing the investigation of the interaction between peroxisomes and microtubules and the quantification of their migration modes with high precision. The HMM allowed us to compare three different experimental conditions with different properties of the microtubule-assisted directed peroxisome migration: wild-type HEK 293 cells (*norm* condition), Nocodazole-treated cells (*noc* condition) with a disturbed integrity of the microtubule, and cells with the *PEX14* gene knockout (*KO PEX14* condition), diminishing the microtubule-interaction of peroxisomes via *PEX14*. While the *norm* condition is a control for highlighting both migration modes and possible switching, the microtubule-interaction of peroxisomes is diminished and thus the directed movement disturbed in the latter two conditions. Standard analysis using mean squared displacement (MSD) analysis was not sufficient to reveal any differences in peroxisome migration between the conditions. Therefore, we introduced the HMM, which was validated using synthetic data to better describe the migration modes. Finally, we applied the HMM to the experimental data to quantify differences between conditions and use the model's parameters to hypothesize why the observed differences occur. The analysis revealed that the knockout of *PEX14* led to a lower probability of directed movement yet not to its complete removal, highlighting alternative microtubule-binding mechanisms besides through *PEX14*. Nocodazole treatment did affect the directed movement, but it was associated with changes in the spatial structure of microtubules rather than due to alterations in peroxisome binding properties.

## Experimental data and computational models

### Cell preparation and imaging

HEK 293 (ATCC, VA, USA) and HEK KO PEX14^23^ cells were maintained in a culture medium consisting of DMEM with 4500 mg glucose/L, 110 mg sodium pyruvate/L supplemented with 10% fetal calf serum, glutamine (2 mM) and penicillin–streptomycin (1%). The cells were cultured at 37 °C/8.5% CO2. Cells were grown on a #1.5 μ-Dish 35 mm (World Precision Instruments, Sarasota, FL) and transfected with 0.5 µg of the eGFP-PEX26 plasmid using Lipofectamine 2000 transfection reagent (Invitrogene, Carlsbad, USA). Imaging of the cells was performed 48 h after transfection. For measurement, the culture medium was substituted with L-15 (Leibovitz's) medium (Thermo Fisher Scientific, Waltham, MA) and placed on the microscope for data acquisition for no longer than 1 h. Each sample was kept in culture medium in the incubator until the measurement started.

For the Nocodazole treatment, the drug was added to the cell culture medium at a final concentration of 10 µM 1 h prior to imaging, while the untreated norm condition was treated with DMSO (1 µl/ml), the solvent for the Nocodazole. With this control, the effect of the DMSO could be excluded.

The eGFP-PEX26 plasmid was created by inserting the eGFP-PEX26 fusion sequence behind the IRES site of the pIRES2 vector. With this construct, a moderate expression level could be reached, as a high overexpression of *PEX26* can cause a mitochondrial mislocalization of the protein. eGFP-PEX26 (2-305) was amplified from the *PEX26* (2-305) in pEGFP-C1 plasmid described in^24^ with the primer pair RE5851: GATCTGTACAAGTCCGGACTCAGATCTAAGAG and RE6035: TCAGTCACGGATGCGGAGCTGG and was integrated into the pIRES2 vector with the restriction enzymes BsrGI and NotI.

The 5-min movies of two-dimensional image frames were taken on a Zeiss Cell Observer Spinning-disc microscope based on a Yokogawa CSU-X1M 5000 Dual Cam Spinning disc unit and a Hamamatsu Orca Flash4.0 V2 sCMOS microscope with a 63x/1.4 Oli M27 objective from Zeiss. Images were acquired with an exposure time of 90 ms in triggered imaging mode to enable a frame rate of $1/{\Delta }t$ with time step ${\Delta }t = 0.1$ s. We tracked the peroxisomes using the ImageJ plugin TrackMate^25^. For TrackMate we used an estimated object diameter of 600 nm, as we know from a morphological STED study^23^ that the size of peroxisomes is between 130 nm to a maximum of 650 nm in diameter and a mean size of 330 ± 125 nm. Furthermore, we set the gap-closing distance to 1 µm and used the settings for “simple LAP tracker”. We represented each track with a vector ${\varvec{X}}_{i} = \left[ {{\varvec{x}}_{i,0} , \ldots , {\varvec{x}}_{{i,T_{max} }} } \right]$, where each entry is a two dimensional spatial position ${\varvec{x}}_{i,t} = \left[ {x_{i,t} , y_{i,t} } \right]$ of peroxisome $i$ at time $t$, from time 0 to *T*_*max*_. Position is measured in μm and time in seconds.

### Experimental conditions and data amount

Tracks were obtained from cells from three experimental conditions, as described in detail above. The first condition was the unaltered, wild-type HEK 293 cells, referred to as the *norm* condition, and these cells served as a control against the other conditions. The second group of cells was treated with Nocodazole. This drug affects microtubule polymerization, which fractures the microtubular network^26,27^, giving us a cell condition that we refer to as *noc. PEX14* is part of the peroxisomal import machinery, essential for importing peroxisomal matrix proteins. Therefore, deleting this protein leads to a loss of peroxisomal import in these cells. The resulting "empty" peroxisomes, also referred to as "ghost peroxisomes", were also efficiently labeled by expression of eGFP-PEX26, as the location of this peroxisomal membrane protein is independent of the import of matrix proteins. We will refer to this condition as *KO PEX14*. We analyzed tracks from 193 cells from *norm* condition (321,012 tracks), 130 cells from the *noc* condition (256,678 tracks), and 131 cells from *KO PEX14* (51,267 tracks). Data was collected from six independent biological replicates. All data can be downloaded at https://asbdata.hki-jena.de/SvenssonEtAl_Peroxisomes.

### Mean squared displacement

The mean squared displacement (MSD) is calculated with the standard expression $MSD\left( t \right) = \frac{1}{N}\mathop \sum \limits_{i = 1}^{N} \left( {{\varvec{x}}_{i,t} - {\varvec{x}}_{i,0} } \right)^{2}$. We grouped tracks from each individual cell to calculate an $MSD\left( t \right)$ for each cell. To avoid short tracks influencing the MSD too much we required a track to be tracked for at least 5 s (50 time steps). To check for anomalous migration modes, we fit the MSD to the power law $MSD\left( t \right)\sim t^{{\alpha_{MSD} }}$ using scikit-learn’s linear regression tool^28^ after log-transform of the power law.

### Hidden Markov model formulation and implementation

**Figure 1:**
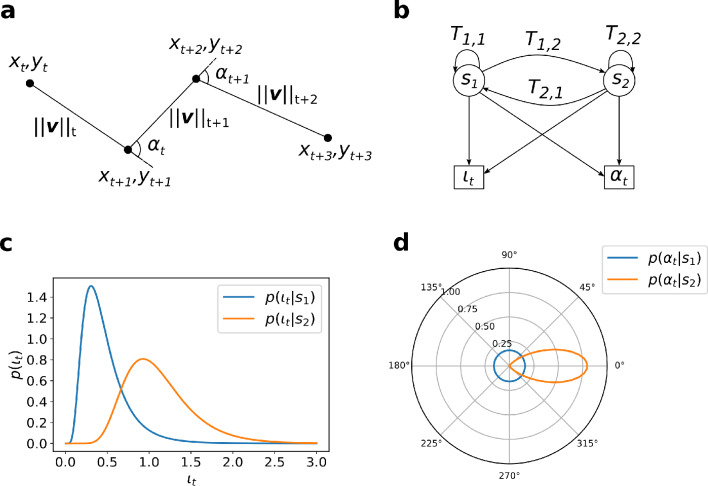
Overview of the HMM formulation. (**a**) Piece of a generic track that visualizes how a series of coordinates convert to the observables instantaneous speed ${\left|\left|{\varvec{v}}\right|\right|}_{t}$ and turning angle ${\alpha }_{t}$. (**b**) Graphical representation of the HMM with states ${s}_{1}$ (random migration mode) and ${s}_{2}$ (directed migration), transition probabilities ${T}_{\mathrm{1,2}}$ and ${T}_{\mathrm{2,1}}$ between the two states, and conversion into the parameters ${\iota }_{t}$ and ${\alpha }_{t}$. (**c**) Representative probability density functions (pdfs) $p\left({\iota }_{t}|{s}_{i}\right)$ for the dimensionless instantaneous speed ${\iota }_{t}$ distributions of peroxisomes in the respective states ${s}_{1}$ and ${s}_{2}$ (Eq. 2) calculated for parameters ${\mu }_{\iota ,1}=-0.93$, ${\mu }_{\iota ,2}=0.06$, ${\sigma }_{\iota ,1}=0.72$ and ${\sigma }_{\iota ,1}=0.50$. (**d**) The pdfs $p\left(\alpha |{s}_{i}\right)$ for the turning angle and ${\alpha }_{t}$ distributions in the respective states ${s}_{1}$ and ${s}_{2}$ calculated from Eqs. 3 and 4 with ${\sigma }_{\alpha ,2}=26^\circ$.

From the spatial positions ${\varvec{x}}_{i,t} = \left[ {x_{i,t} , y_{i,t} } \right]$ over time $t$ of the two-dimensional peroxisome tracks, we extracted the relative turning angle, $\alpha_{t}$, and the instantaneous speed of the peroxisome, $\left| {\left| {\varvec{v}} \right|} \right|_{t} = \frac{{\sqrt {\left( {x_{i,t + 1} - x_{i,t} } \right)^{2} + \left( {y_{i,t + 1} - y_{i,t} } \right)^{2} } }}{{{\Delta }t}}$. To be able to use the log-normal distribution for the speed, we define the dimensionless speed $\iota_{t} = \frac{{\left| {\left| {\varvec{v}} \right|} \right|_{t} }}{{1 \;\upmu {\text{m/s}}}}$^29^ to be able to convert the speed with the natural logarithm. In Fig. 1a, we have illustrated how we converted the coordinates to the observables instantaneous speed and relative turning angle, ${\varvec{o}}_{t} = \left[ {\iota_{t} ,\alpha_{t} } \right]$, which we then used in our HMM.

The hidden states were denoted by $s_{1}$ and $s_{2}$, which represents the random mode of peroxisome migration not attached to the microtubule and the directed migration of attached peroxisomes. We assumed that the instantaneous speed and turning angle were conditionally independent when conditioned on each state, allowing us to separate the joint probability distribution function (pdf) $p\left( {{\varvec{o}}_{t} {|}s_{i} } \right)$ of the distribution of value pairs ${\varvec{o}}_{t} = \left[ {\iota_{t} ,\alpha_{t} } \right]$ in the respective states $s_{1}$ and $s_{2}$, into the individual pdfs $p\left( {\iota_{t} {|}s_{i} } \right)$ and $p\left( {\alpha_{t} {|}s_{i} } \right)$, i.e.

1 $$p\left( {{\varvec{o}}_{t} {|}s_{i} } \right) = p\left( {\iota_{t} {|}s_{i} } \right)p\left( {\alpha_{t} {|}s_{i} } \right)\quad {\text{for}}\quad i \in \left[ {1,2} \right].$$

Figure 1b is a graphical overview of the HMM, where the variables $T_{i,j} \left( {i,j \in \left[ {1,2} \right]} \right)$ indicate the transition probabilities between hidden states at each time step. For the pdf of the distribution of dimensionless instantaneous speed $\iota_{t}$ of the peroxisomes, we used the log-normal distribution for both states but with independent parameters $\mu_{\iota ,i}$ and $\sigma_{\iota , i}$ (i = 1 or 2, compare Fig. 1c).

2 $$p\left( {\iota_{t} {|}s_{i} } \right) = lognorm\left( {\mu_{\iota ,i} ,\sigma_{\iota , i} } \right)$$

The use of a log-normal distribution avoids the issue with negative speeds, which would be accompanied by a normal distribution while preserving the possibility of using expectation maximization to fit the HMM. In Supplementary Figs. S1a and b, we show that the shapes of the speed distribution for both states are indeed well described by a log-normal distribution. The parameters $\mu_{\iota ,i}$ and $\sigma_{\iota , i}$ are the dimensionless parameters of the log-normal distribution as they are the mean and standard deviation of the logarithm of the instantaneous speed^29^.

For the pdfs of the distribution of values of turning angle, we applied a uniform distribution and a normal distribution (centered at zero and with a standard deviation $\sigma_{\alpha ,2}$) for the random and directed migration modes, respectively:

3 $$p\left( {\alpha_{t} {|}s_{1} } \right) = uniform\left( {0^\circ ,360^\circ } \right),$$

4 $$p\left( {\alpha_{t} {|}s_{2} } \right) = normal\left( {0,\sigma_{\alpha ,2} } \right).$$

Figure 1c, d visualize the pdfs for $\alpha_{t}$ and $\iota_{t}$ for the two states for representative mean and standard deviation values. In Supplementary Figs. S1c–f, we visualized the turning angle distributions for each state to motivate the accurate assumption of conditional independence between speed and turning angles and the distribution of angles.

To determine the most likely state given an observation ${\varvec{o}}_{t}$, we used Bayes' theorem

5 $$p\left( {s_{i} {|}{\varvec{o}}_{t} } \right) = \frac{{p\left( {{\varvec{o}}_{t} {|}s_{i} } \right)p\left( {s_{i} } \right)}}{{\mathop \sum \nolimits_{i = 1}^{2} p\left( {{\varvec{o}}_{t} {|}s_{i} } \right)p\left( {s_{i} } \right)}},$$

with the pdfs $p\left( {{\varvec{o}}_{t} {|}s_{i} } \right)$ given in Eqs. (1)–(4), and the prior probability $p\left( {s_{i} } \right)$ that a track is in state $s_{i}$ defined as

6 $$p\left( {s_{i} } \right) = \pi_{i} , {\text{with }}\mathop \sum \limits_{i = 1}^{2} \pi_{i} = 1.$$

Here, the parameters $\pi_{1}$ and $\pi_{2}$ make up the vector ${\varvec{\pi}} = \left[ {\pi_{1} ,\user2{ }\pi_{2} } \right]$ and express the overall proportion of steps that the tracks spend in state $s_{1}$ and $s_{2}$, respectively.

### Parameter fitting and state inference

The parameters of the HMM (${\varvec{\mu}}_{\iota ,t} , {\varvec{\sigma}}_{\iota ,t} , \sigma_{\alpha ,2}, {\varvec{\pi}}, {\varvec{T}}$) were fitted using the scaled Baum-Welch algorithm, which finds the maximum likelihood values for the parameters given data^30^. The maximum likelihood solution was found using a scaled version of Expectation Maximization. The inference of states based on observables was performed by the Viterbi algorithm^31^.

### Model for generation of synthetic data

In addition to the experimental data, we also generated synthetic data of peroxisome tracks. We let the simulated tracks switch between the random migration mode and directed migration mode with probabilities of $T_{1,2} = T_{2,1} = 0.1$ at each time step and with initial probabilities of starting in each state of $\pi_{1} = \pi_{2} = 0.5$. We simulated 100 tracks with 200 steps each. For the random migration, we used a discrete version of Ornstein-Uhlenback process and generated new track positions for each timestep ${\Delta }t = 1 \;{\text{s}}$ by calculating

7 $${\varvec{v}}_{t + 1} = \left( {1 - \frac{{{\Delta }t}}{\tau }} \right){\varvec{v}}_{t} + \sqrt {{\Delta }t} \frac{{\sqrt {2D} }}{\tau }\epsilon_{t} ,$$

8 $${\varvec{x}}_{t + 1} = {\varvec{x}}_{t} + {\Delta }t{\varvec{v}}_{t + 1} .$$

Here, ${\varvec{v}}_{t}$ is the instantaneous velocity, $\tau$ is the persistence of the velocity, D is the diffusion coefficient of the random walk process, and $\epsilon_{t}$ considers noise. We set the parameters to $\tau = 1.5 s$, $D = 5 m^{2} /s$ and $\epsilon_{t}$ = $normal\left( {0,1} \right)$ distributed noise to get a qualitatively similar migration behavior as we see in the peroxisome tracks.

The dynamics of the directed migration mode were calculated by

9 $$\left| {\left| {\varvec{v}} \right|} \right|_{t + 1} = normal\left( {10, 1} \right),$$

10 $${\varvec{v}}_{t + 1} = \frac{{{\varvec{v}}_{t} }}{{\left| {\left| {{\varvec{v}}_{t} } \right|} \right|}}\left| {\left| {\varvec{v}} \right|} \right|_{t + 1} + \epsilon_{t} ,$$

11 $${\varvec{x}}_{t + 1} = {\varvec{x}}_{t} + {\Delta }t{\varvec{v}}_{t + 1} ,$$

where $\left| {\left| {\varvec{v}} \right|} \right|_{t}$ is the instantaneous speed drawn from a normal distribution around $10{ }\;{\text{m/s }}$ with a standard deviation of $1{ }\;{\text{m/s}}$.

### Validation measures

We used the performance measures recall, precision and F1 to evaluate how well the HMM can infer states given the observables from the synthetic data. We defined the directed state, $s_{2}$, as the positive state and can thereby count the number of true positives ($TP$), false positives ($FP$), true negatives ($TN$) and false negatives ($FN$) that the inference of states by the HMM gave us. We then used this to calculate the measures

12 $$recall = \frac{TP}{{TP + FN}},$$

13 $$precision = \frac{TP}{{TP + FP}},$$

14 $$F1 = \frac{precision \cdot recall}{{precision + recall}}.$$

### Significance testing

Significance testing was performed using a two-sided Student's t-test as implemented in SciPy's stats-library^32^. To control for multiple comparisons, we used Bonferroni correction^33^ to turn the raw *p* value, $p$, into a corrected value, $q$.

### Model implementation

MSD analysis and HMM were implemented in Python 3.7 and the code is available on GitHub (https://github.com/applied-systems-biology/Peroxisome_HMM). Numpy^34^ was used for both the calculations for the MSD and HMM analysis. For the speed-up of the HMM, we used Numba^35^.

## Results

We recorded migration tracks of individual peroxisomes (fluorescently tagged via eGFP-PEX26) in live HEK 293 cells under physiological conditions using two-dimensional spinning disc fluorescence microscopy at an acquisition rate of 10 frames/s and for three experimental conditions with respect to peroxisome-microtubule interactions: (1) *norm*: untreated cells with an unperturbed microtubular network; (2) *noc*: cells treated with Nocodazole to disassemble the microtubular network and thereby presumably eliminating almost all directed migration; and (3) *KO PEX14*: HEK KO PEX14 cells where the peroxisomal membrane protein *PEX14* that is supposed to be responsible for microtubule attachment of peroxisomes, was knocked out using CRISPR. In this condition the peroxisomes are less abundant compared to the *norm* and *noc* condition, which can be seen in the example movies S6 (*norm*) and S8 (*KO PEX14*), as well as in the statistical analysis of the peroxisome density in the analyzed cells in Supplementary Fig. S2.

### Mean square displacement analysis does not reveal differences between experimental conditions

**Figure 2:**
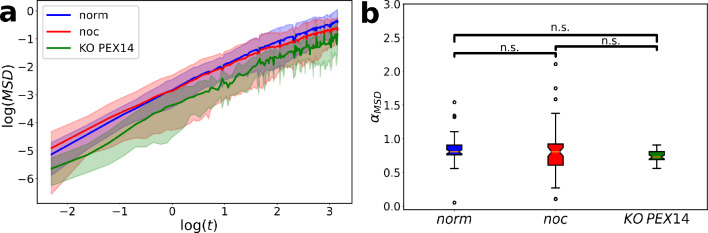
MSD analysis of peroxisome tracks across conditions. (**a**) MSD curves for the three conditions display similar dynamics across conditions. Shaded regions indicate standard deviation across cells in each condition. (**b**) Fitted values ${\alpha }_{MSD}$ of the exponent in $MSD\sim {t}^{{\alpha }_{MSD}}$ as quantification of potential anomalous diffusion dynamics, revealing similar values across conditions.

A standard method of track analysis^36^ that has already been used to analyze peroxisome movement along microtubules in *Drosophila S2* cells is the MSD calculation^21^. We chose all tracks that were tracked for at least 5 s (50 time steps) and calculated the MSD between 0 and 20 s for each experimental condition (*norm*, *noc* and *KO PEX14*), as shown in Fig. 2a.

The shaded region in Fig. 2a indicates the standard deviation of the $MSD\left( t \right)$ between cells. Qualitatively, it is clear from Fig. 2a that the dynamics were similar across the conditions. To get a more quantitative representation of the MSD dynamics, we also fitted an exponential model to $MSD\left( t \right)$. We used the power-law formulation with the anomaly parameter $\alpha_{MSD}$ for detecting anomalous diffusion,

15 $$MSD\left( t \right)\sim t^{{\alpha_{MSD} }} ,$$

which regularly has been used to differentiate superdiffusive ($\alpha_{MSD} > 1$) and subdiffusive ($0 < \alpha_{MSD} < 1$) migration from pure Brownian motion ($\alpha_{MSD} = 1$)^37^. We fitted $\alpha_{MSD}$ for each cell across the three conditions and, as can be seen in Fig. 2b, all conditions did exhibit slight subdiffusive motion ($\alpha_{MSD}$ ≈ 0.80) with no significant difference between conditions. It is well perceived, that organelle diffusion inside the cellular cytoplasm is on average subdiffusive due to interactions with other objects, such as the microtubular network^38,39^ or the endoplasmic reticulum, mitochondria and lipid droplets^12,40^. For mixed migration systems known to have active transport, superdiffusive migration has been observed on longer timescales^21,41^, but we noticed no such trends based on the analysis of $MSD\left( t \right)$ (see Fig. 2a). In conclusion, standard MSD analysis of the tracks did not give any indication of the directed movement or any difference between the experimental conditions. The main outcome of the MSD analysis was that the peroxisomes were moving slightly subdiffusive.

### Hidden Markov Model fitting performs with high accuracy on simulated data

The results from the previous investigation methods of peroxisome tracks, e.g., MSD measurements, made us expect that the directed migration mode would be a rare occurrence and would typically only consist of a few consecutive time steps. Therefore, we employed an HMM fitting approach with the two states $s_{1}$ and $s_{2}$ i.e., random and directed migration, as detailed in the Experimental data and computational models section.

**Figure 3:**
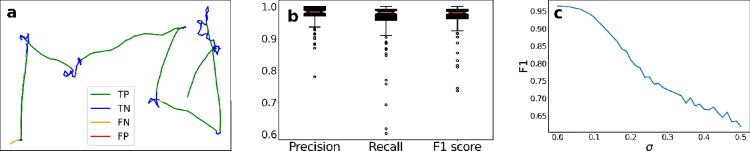
HMM validation on synthetic data. (**a**) Comparison of ground truth states and predicted states after fitting the parameters with Baum–Welch and determining the state of each time step using the Viterbi algorithm. (**b**) Performance measure of accurate state assignment using the Viterbi algorithm for each time step. (**c**) The effect on the F1 measure when disturbing the fitted parameter ${{\varvec{\mu}}}_{\iota }$ by adding normal distributed noise with zero mean and standard deviation $\sigma$ before predicting the states using the Viterbi algorithm.

To test the accuracy of the HMM fitting, we first employed it to simulated data. Figure 3a shows a representative simulated track generated from our model for synthetic data [see Eqs. (7)–(11)]. We fitted all parameters of the HMM to each track using the Baum–Welch algorithm and then determined the track's state at each time point with the Viterbi algorithm (see section “Parameter fitting and state inference”). The color code in Fig. 3a indicate whether a single time step of the example track was determined to be a true positive (*TP*), true negative (*TN*), false positive (*FP*) or false negative (*FN*) according to the definitions in the Validation measures section after inferring the track states using the Viterbi algorithm. We see that inference mistakes are mostly occurring when close to state switches from directed to random migration or vice versa. Across all 100 simulated tracks, the HMM yielded correct predictions of on average 97.3% per track. Figure 3b reveals the high performance in terms of the precision, recall and F1-score across the simulated tracks, where we defined the state $s_{2}$ to be the positive condition (see section Validation measures). It should be noted that the errors are balanced between false positives and false negatives as there is a small difference between $recall$ and $precision$, which indicates that there is no bias in the determination of the states.

To test the sensitivity of the fitted parameters, we disturbed the fitted values of ${\varvec{\mu}}_{\iota }$ by adding normal distributed noise with zero mean and varying the standard deviation $\sigma$ between 0 and 0.5. Then we measured the F1-score as a function of the strength of the added noise as indicated by $\sigma$ and plotted this in Fig. 3c. As we can see in Fig. 3c, is the state inference reasonably stable for small disturbances of ${\varvec{\mu}}_{\iota }$ where $\sigma < 0.1$. The average fitted values for the speed parameter of simulated data were ${\varvec{\mu}}_{\iota } = \left[ {1.22, 2.30} \right]$, which means that added noise with $\sigma = 0.1$ translates to to $5 - 10$% disturbance of the parameter values on average. For $\sigma > 0.1$ we see in Fig. 3c that the F1-score fall of quickly.

To compare our model with already published state-of-the-art HMM models, we used the HMM Bayes approach published by Monnier et al.^42^. This method is more flexible as it allows for scanning tracks for an arbitrary number of states with the underlying assumption is that the track has inherently different diffusivity in the states rather than directionality. The tracks analyzed in the original publication had mixed migration modes that were either Brownian motion or Brownian motion with a directed flow. Therefore, those tracks were quantitatively similar to our tracks. However, the HMM Bayes approach predicted the state of each track on average on 76% of the time steps from our simulated data. The model had a high precision, i.e. if the model suggested directed motion, this is correct in about 95% of the cases, but a low recall of around 55%, see Supplementary Fig. S3. For HMM Bayes, we limited the number of possible states to two, and to process one of our 100 steps long, simulated tracks took an average of 48 s on a Linux server with 80 Intel(R) Xeon(R) CPU E5-2698 v4 cores. On the same machine, fitting all parameters and predicting the states of each track with our HMM took an average of 0.8 s per track.

### Hidden Markov Model fitting reveals peroxisome speed distribution

**Figure 4:**
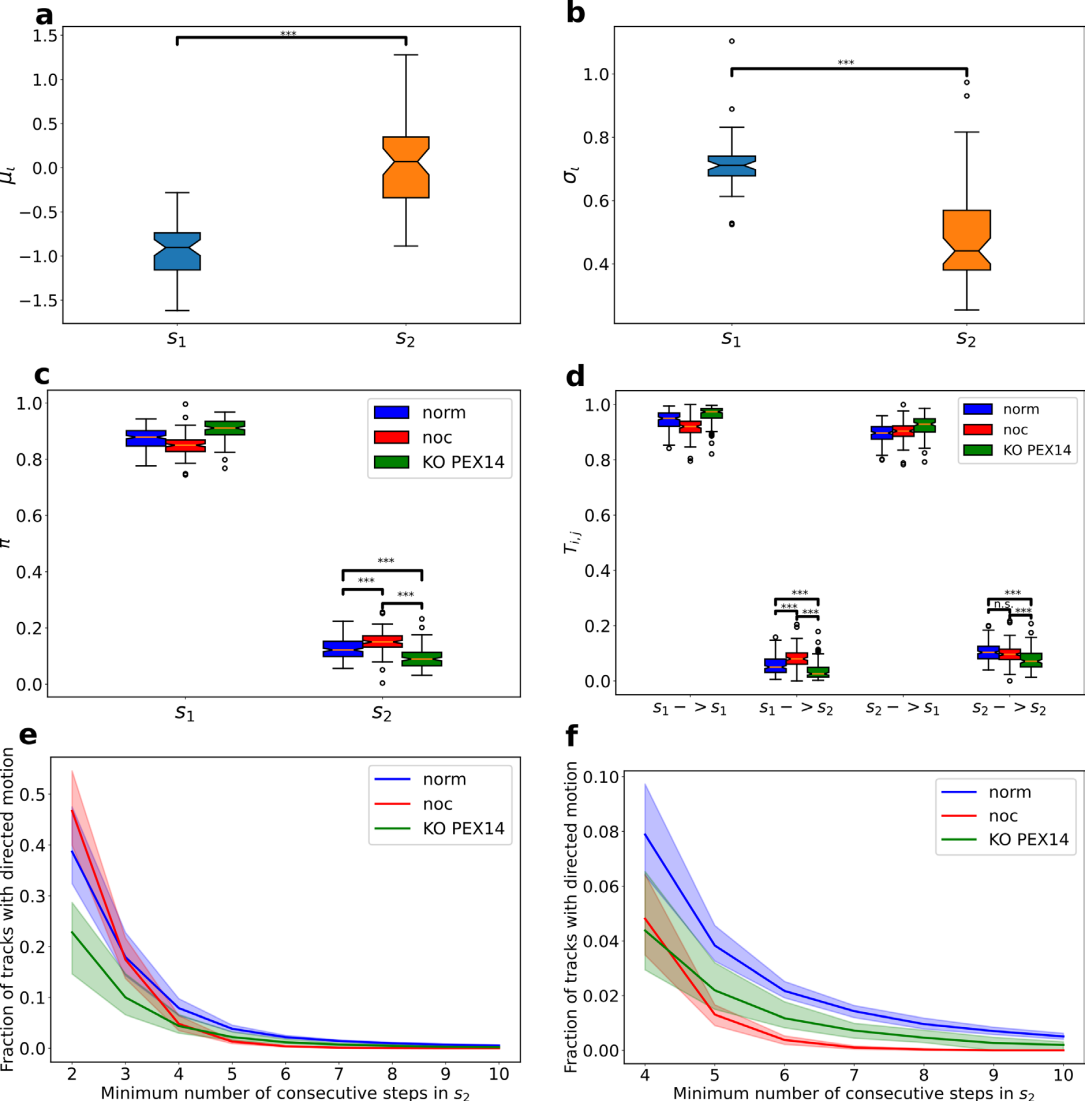
Fitting of HMM parameters. Values of ${\mu }_{\iota }$ (**a**) and ${\sigma }_{\iota }$ (**b**) after fitting the parameters to 52 manually selected norm tracks with mixed migration modes. (**c**) The ratio of steps in states $s_{1}$ and $s_{2}$ expressed by the values of $\pi_{i}$ from the HMM after applying it to all tracks. (**d**) The values of the transition matrix after fitting it to all tracks. (**e**, **f**) The ratio of tracks showing consecutive directed steps as a function of the minimum number of consecutive steps required. In (**f**) we have a zoomed-in version of the curves in (**e**) to highlight the abolishment of tracks with more than six consecutive directed steps in the *noc* condition. Shaded regions indicate 95% confidence intervals obtained by bootstrapping. Stars represent the significance level after Bonferroni corrected *p* value: n.s.: q > 0.05, ***: q < 0.001. For panels (**c**) and (**d**), significance is only indicated in one of the states. The same significance pattern naturally holds for the other state since all $\pi_{i}$ and the rows in ${\varvec{T}}$ add up to one.

The results from previous investigation methods of peroxisome tracks, e.g., MSD measurements, made us expect that the directed migration mode would be a rare occurrence and typically only consist of a few consecutive time steps. Therefore, we manually selected 52 tracks from seven cells in the *norm* condition that exhibited a mix of both states. In Supplementary Fig. S4a, we give an example of such a track, and in Fig. 4a, b, we show the values of $\mu_{\iota ,i}$ and $\sigma_{\iota ,i}$, as extracted from fitting Eq. (2) to the pdf of the instantaneous speed $\iota_{i,t}$ distribution using our HMM approach with the two states $s_{1}$ and $s_{2}$, i.e. random and directed migration, respectively (compare Eqs. (1–2) and Fig. 1c). During determination of $\mu_{\iota ,i}$ and $\sigma_{\iota ,i}$ we allowed all parameters of the HMM to vary while fitted by the scaled Baum-Welch algorithm^30,31^. On average, we got ${\varvec{\mu}}_{\iota } = \left[ { - 0.93, 0.06} \right]$ and ${\varvec{\sigma}}_{\iota } = \left[ {0.72, 0.50} \right]$, which transforms to the expectation values of instantaneous speeds $E\left[ {\left| {\left| {\varvec{v}} \right|} \right|_{t} {|}s_{1} } \right] = 0.5$ μm/s and $E\left[ {\left| {\left| {\varvec{v}} \right|} \right|_{t} {|}s_{2} } \right] = 1.2$ μm/s through the calculation $\overline{\user2{\iota }} = e^{{{\varvec{\mu}}_{\iota } + \frac{{{\varvec{\sigma}}_{\iota }^{2} }}{2}}}$^43^. These values already highlight that the average speed for the directed migration was more than double the speed of the random migration of the peroxisomes. Instead of determining the expectation values, we also calculated the average instantaneous speed across all tracks from the seven cells of the *norm* condition that we used for fitting the migration parameters of the HMM, resulting in $E\left[ {\left| {\left| {\varvec{v}} \right|} \right|_{t} } \right] = 0.58$ μm/s. This is considerably closer to the speed of random migration than directed migration, which is unsurprising as we expect that this migration mode is dominant.

The estimation of peroxisome speed is naturally highly dependent on the temporal resolution of the microscope. To demonstrate this, we downsampled the track data by only taking every *k*th time step and recalculated the estimated peroxisome speed. At double the time step, ${\Delta }t = 0.2$ s, we saw that the estimate was reduced to $E\left[ {\left| {\left| {\varvec{v}} \right|} \right|_{t} } \right] = 0.38$ μm/s; further downsampling to ${\Delta }t = 2$ s yielded an estimated speed value of only $E\left[ {\left| {\left| {\varvec{v}} \right|} \right|_{t} } \right] = 0.09$ μm/s. In Supplementary Fig. S5, we have plotted the estimated instantaneous speed as a function of sampling frequency.

### HMM parameter analysis reveals peroxisome dynamics after fitting

In the previous section, we described how we determined all underlying parameters ($\mu_{\iota ,i} { }$ and $\sigma_{\iota ,i}$) for the migration of peroxisomes in states ${ }s_{1}$ and $s_{2}$ by fitting $p\left( {\iota_{t} {|}s_{i} } \right){ }$ using the manually selected tracks of the norm condition. We have now fixed these parameters and scanned all tracks from all three conditions. Specifically, we fitted ${\varvec{\pi}} = \left[ {\pi_{1} ,{ }\pi_{2} } \right]$ and $${\varvec{T}} = \left[ {\begin{array}{*{20}c} {T_{1,1} } & {T_{1,2} } \\ {T_{2,1} } & {T_{2,2} } \\ \end{array} } \right]$$ for each track using the Baum-Welch algorithm and determined the state of each track step using the Viterbi algorithm^31^. An example of a color-coded track after state determination is plotted in Supplementary Fig. 4b. Further, Fig. 4c, d depict the values of ${\varvec{\pi}}$ and ${\varvec{T}}$ across the three conditions after calculating the average for each cell.

### Long, directed migration is abolished in the *noc* condition

Figure 4c highlights that the *noc* condition had the highest probability,${\pi }_{2}$, of being in the directed migration state ${s}_{2}$. Further, the values of the transition matrix ${\varvec{T}}$ (Fig. 4d) revealed that the *noc* condition was more likely than the *norm* condition to transfer from ${s}_{1}$ to ${s}_{2}$. The reverse transfer from ${s}_{2}$ to ${s}_{1}$ did not show any significant difference between *noc* and *norm* conditions, suggesting that the higher overall probability for *noc* to be in ${s}_{2}$ was due to a higher transfer and thus microtubule-binding frequency. To further gauge the mechanism of peroxisome binding to the microtubule, we considered the distribution of the ratio of tracks that have a minimum number of consecutive steps in the state ${s}_{2}$, i.e. of directed migration. Figure 4e highlights the larger ratio of tracks in state ${s}_{2}$ for the *noc* compared to the *norm* condition, but this was only the case for one or two consecutive steps in ${s}_{2}$, i.e. only for very short directed migration events, and the ratio fell off quickly. The frequency of longer directed migration events (three or more steps in ${s}_{2}$) became significantly less for *noc* tracks than for the *norm* condition.

### Long, directed migration is decreased in the *KO PEX14* condition

As highlighted, we expected the directed migration to basically diminish in the *KO PEX14* condition, since *PEX14* plays a vital role in the binding of peroxisomes to microtubules^10,15^, and its knock-out should thus impede microtubule-assisted movement. Indeed, the values of ${\varvec{\pi}}$ in Fig. 4c reveal the least likely condition to be in $s_{2}$ for the *KO PEX14* condition. Further, the values of the transition matrix $T$ (Fig. 4d) depict that for the *KO PEX14* condition it was most unlikely to transfer from random mode ($s_{1}$) to directed migration ($s_{2}$) as well as most likely to transfer from directed ($s_{2}$) to random migration ($s_{1}$). Figure 4e, f show that the ratio of *KO PEX14* tracks with consecutive steps in $s_{2}$ was consistently below *norm* tracks and only until five consecutive steps below the *noc* condition, thereafter getting above in a similar way as discussed before for *noc* and *norm* conditions. We also noted that independent of condition, having ten consecutive steps in $s_{2}$ was extremely rare, i.e., less than 1% of the tracks stayed in $s_{2}$ for such a long time (Fig. 4f). Still, migrated movement was not completely eradicated.

## Discussion

We analyzed peroxisome tracks of high temporal and spatial resolution with the goal of investigating the microtubule-dependent movement of peroxisomes in live HEK 293 cells. Published data has described two different modes of movement for peroxisomes, a vibrational movement on the one hand and a fast, directed, microtubule-based movement on the other hand^9,11–16,19,21^. However, these experiments were still limited with respect to for example total track numbers, automated and non-arbitrary data analysis, temporal and spatial resolution, and detection of possible mode-switching within a single track. Further, they did not fully explore whether microtubule-interaction and thus microtubule-assisted migration is solely dependent on the peroxisomal protein *PEX14* we aimed to describe these two types of movement in more detail, with greater statistical power and higher temporal resolution than the previous studies. To reach this goal, we imaged at least 130 cells, resulting in at least 50,000 tracks of moving peroxisomes for each condition, recorded with a high temporal resolution of 10 frames/s. This large amount of data enabled us to quantify the movement of all peroxisomes in a population of cells, while previous studies primarily focused on the analysis of representative tracks from single cells. We analyzed peroxisome movement under different conditions without (*norm* condition) and with disturbance of the directed microtubule-assisted migration mode (*noc* condition: diminished integrity of the microtubule through Nocadozole treatment; and *KO PEX14* condition: through *PEX14* gene knockout). We visually observed how peroxisomes made fast, straight migrations, and from inspection of the video data (Supplementary Videos S6–S8) it seemed that this behavior was more common in the *norm* condition than in the other ones. Our initial attempts to quantify the movement behavior of peroxisomes within our large data set using standard methods, exemplified by MSD analysis, were unsuccessful in showing any superdiffusive movement or differences between conditions, yet in an automated and unbiased way. Here, the problem is that the directed movement is extremely rare compared to the vibrational movement, so it is necessary to find a way to distinguish between these two modes for each track. We see that especially longer consecutive directed motion is extremely rare even in the *norm* conditioning, indicating that using for example a sliding window MSD analysis^44,45^ would need to be very precisely tuned and it could still be that the directed section would be too short to give an impact on the MSD. Facing these problems, we decided for an HMM to distinguish between the two different states of movement, as HMMs have already been used to investigate single-particle tracks to analyze different diffusive states^42,46–50^. The use of HMMs allowed for the investigation of tracks that swap migration modes, while standard analysis methods like MSD or persistent random walks assume that each track is in a single migration mode throughout its duration^51^. With this approach, we were able to analyze the change of the different modes of movement of a single peroxisome within one track. This has not been done before and allowed us to study the differences in switching from one mode to the other for the first time. However, most established HMMs focus on finding different Brownian motion classes^47,49,50,52^. In our case, we were explicitly looking for two separate states of migration: random migration, when a peroxisome is not attached to the microtubular network, and directed migration, when a peroxisome is migrating along microtubules. Therefore, the more general frameworks that allow for anomalous diffusion, like Bayes HMM^42^ or NOBIAS^48^, are too general as the states are well known. We demonstrated this by trying the Bayes HMM package from Monnier et al.^42^ on our simulated data, finding that this approach was several orders of magnitude slower and less accurate in determining the hidden state used to generate the data. Bayes HMM or NOBIAS are more flexible tools than our HMM as they can scan tracks with an arbitrary number of hidden states based on the track diffusivity. They would, therefore, be preferred if we were scanning tracks for general anomalous migration, like strongly confined migration. However, as we were looking for the specific states of directed and non-directed migration in many tracks, our model was performing considerably better on this dataset.

We created an HMM where one of the states specifically represented the directed migration mode. Compared with many earlier studies that found these states by manually setting a threshold between a "fast" and a "slow" peroxisomal population^9,13,19^, the HMM has several advantages. Determining such a threshold when one state is much rarer than the other, like in the case of directed versus random migration, is precarious. In other cases, thresholds have been motivated by speed distributions from other studies^19,53^. However, we saw that the estimate of peroxisome speed depends strongly on the experimental settings, such as frame rate (see Supplementary Fig. S4), which is a common limitation in tracking. In the formulation of the HMM, we did not assume anything about the speed of migration in the respective migration mode but only assumed that migration should be directed.

Consequently, all differences in the speed distributions between the states were inferred from the data in an unsupervised way using the scaled Baum–Welch algorithm. The probabilistic nature of the HMM also simultaneously weighed the observables, speed and turning angle for the classification in a more nuanced manner than single thresholds in one dimension can do. We showed that the HMM could detect directed migration in simulated data and for manually selected real data, see Fig. 3 and Supplementary Fig. S4. The first result was that the directed mode was associated with a considerably higher average speed of the peroxisomes. This is consistent with the idea of the active transport of peroxisomes via microtubules.

We then scanned all tracks with more than 50 time steps across our three conditions, *norm*, *noc* and *KO PEX14,* and fitted the parameters ${\varvec{\pi}}$ and ${\varvec{T}}$ to each cell. The parameter ${\varvec{\pi}}$ describes the overall probabilities of a track to be in each of the states $s_{1}$ and $s_{2}$ while the transition matrix ${\varvec{T}}$ gives the transition probabilities between states. The *noc* condition displayed an increased probability of actually being found in the state $s_{2}$ that represents the directed migration (see Fig. 4c). Compared to the *norm* condition, the peroxisomes in *noc* condition cells did not stay in $s_{2}$ for more than three consecutive time steps. It is known that Nocodazole treatment fractures the microtubular network^26,27^, and a common hypothesis so far was that the treatment entirely abolishes directed migration, as deduced by Rapp et al*.*^9^. However, the study by Rapp et al*.* is severely limited by the 30 s between frames used for data acquisition. Given that almost no peroxisomes in the *noc* condition stay in the directed migration state for more than 0.5 s (see Fig. 4f), it is not surprising that Rapp et al. could not observe any directed migration after Nocodazole treatment. We hypothesize that Nocodazole treatment does not affect the binding of peroxisomes to the microtubule, but as there is no continuous network, there is no possibility of directed transport over longer spatial and temporal distances. The fact that peroxisomes are, on average, more likely to be in $s_{2}$ could be explained by the fact that the fractured network makes the interaction between peroxisomes and microtubule fragments more likely because of the increased spatial homogeneity of the fragments compared with a microtubular network.

Previous studies^15^ suggested *PEX14* as an essential compound for peroxisome long-range motility. *PEX14* directly interacts with β-tubulin, a component of microtubules, indicating that it serves as the connecting link for anchoring peroxisomes to microtubules to initiate peroxisomal directional movement^16^. Follow-up studies confirmed the critical role of *PEX14* for peroxisome motility but also demonstrated that peroxisomes can move along MT in *PEX14*-deficient eukaryotic cells^19^. This suggests that other adaptors can substitute the *PEX14* MT-tethering function. One of the possible candidates is Miro1, which connects motor proteins to the surface of peroxisomes and mitochondria^19,54^. Also, other peroxins like Pex1 might be involved in these processes^10,55^.

Consistently, microtubule binding was not completely eliminated in the *KO PEX14* condition, as shown in our analysis. However, tracks from *KO PEX14* cells had a lower probability of ever being in $s_{2}$, see Fig. 4c, and was less likely to enter state $s_{2}$ from $s_{1}$, see Fig. 4d. It could possibly be argued that two or three consecutive steps in the directed migration mode could occur because of randomness, but as we see in Fig. 4d, we have a significantly higher ratio of tracks with six to ten consecutive steps in $s_{2}$ then in the *noc* condition. We, therefore, concluded that even without *PEX14*, peroxisomes could bind to microtubules and stay attached for multiple steps, but the probability of binding is lower than in the *norm* or *noc* condition. This is a different mechanism than seen in the *noc* condition, where the binding is common, but there are very few tracks with more extended consecutive periods in $s_{2}$.

### Supplementary Information

Supplementary Information 1.

Supplementary Video 1.

Supplementary Video 2.

Supplementary Video 3.

## Data Availability

All data can be downloaded at https://asbdata.hki-jena.de/SvenssonEtAl_Peroxisomes and code is available at https://github.com/applied-systems-biology/Peroxisome_HMM.
