# Supplementary material for: Quantitative analysis of peroxisome tracks using a Hidden Markov Model
Source: Sci Rep. 2023 Nov 11;13:19694. doi: 10.1038/s41598-023-46812-7 (PMC10640649; doi:10.1038/s41598-023-46812-7)
Supplement: Supplementary file 1 — Supplementary Information 1. [file 41598_2023_46812_MOESM1_ESM.pdf]

Supplementary Material: Quantitative Analysis of Peroxisome Tracks using a Hidden Markov Model

Carl-Magnus Svensson[a,†], Katharina Reglinski[b,c,d,e,†], Wolfgang Schliebs[f], Ralf Erdmann[f],

Christian Eggeling [*,b,c,d,g], Marc Thilo Figge[*,a,f,h]

[a] Applied Systems Biology, Leibniz Institute for Natural Product Research and Infection Biology - Hans Knöll Institute, Jena, Germany

[b] Leibniz-Institute of Photonic Technologies, Jena, Germany

[c] Institute of Applied Optics and Biophysics, Friedrich-Schiller University Jena, Jena, Germany

[d] MRC Human Immunology Unit, Weatherall Institute of Molecular Medicine, University of Oxford, Oxford, United Kingdom

[e] University Hospital Jena, Jena, Germany

[f] Institute of Biochemistry and Pathobiochemistry, Systems Biochemistry, Ruhr-University Bochum, Bochum, Germany

[g] Jena Center for Soft Matter (JCSM), Jena, Germany

[h] Institute of Microbiology, Faculty of Biological Sciences, Friedrich-Schiller University Jena, Jena, Germany

[†] Authors contributed equally

Corresponding authors: christian.eggeling@uni-jena.de and thilo.figge@hki-jena.de

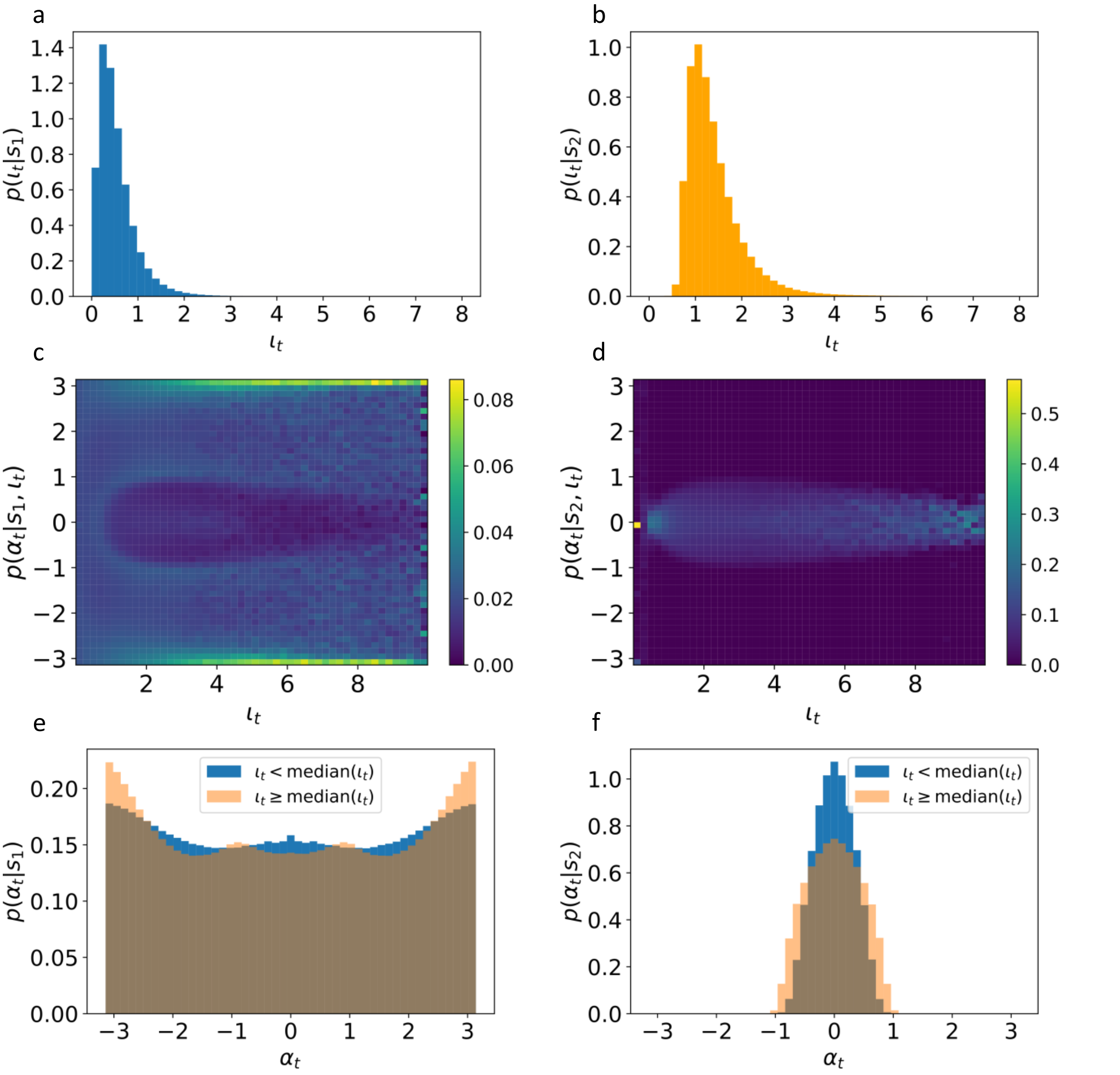

Figure S1: Speed and turning angle distributions of the experimental data. a-b) Distributions of $\boldsymbol{\iota}_t$ for s_1 and s_2 respectively and c-d) distribution of turning angle as a function of speed for s_1 and s_2 respectively. e-f) Turning angle distribution comparisons for low speed and high speed peroxisomes, as determined by the median speed, for s_1 and s_2 respectively.

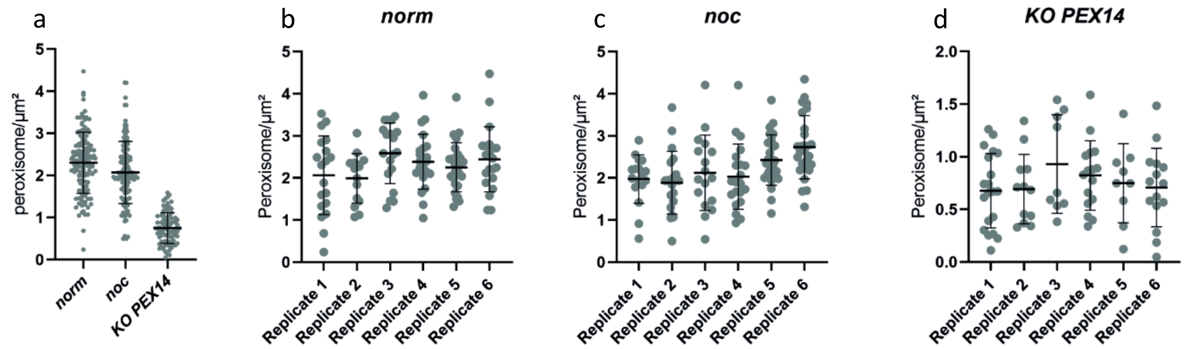

Figure S2: Comparison of biological replicates. a) The peroxisome density for the three conditions *norm, noc* and *KO PEX14.* b-d) Comparison of the peroxisome density across biological replicates for each of the three experimental conditions. Each dot is representing a single cell, shown are mean values with standard deviation.

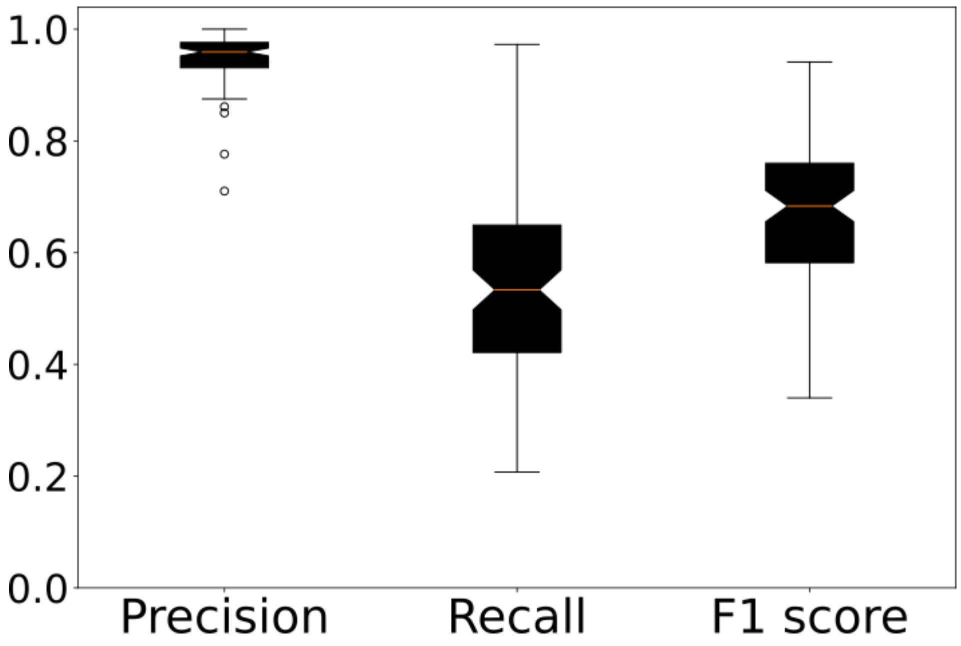

Figure S3: Bayes HMM applied to synthetic data. The precision, recall and F1 score when applying Monneir et al. Bayes HMM [1] to the simulated data. This model is focused more on diffusion than straightness. Therefore, it does not capture the slower directed motion as well as our HMM model.

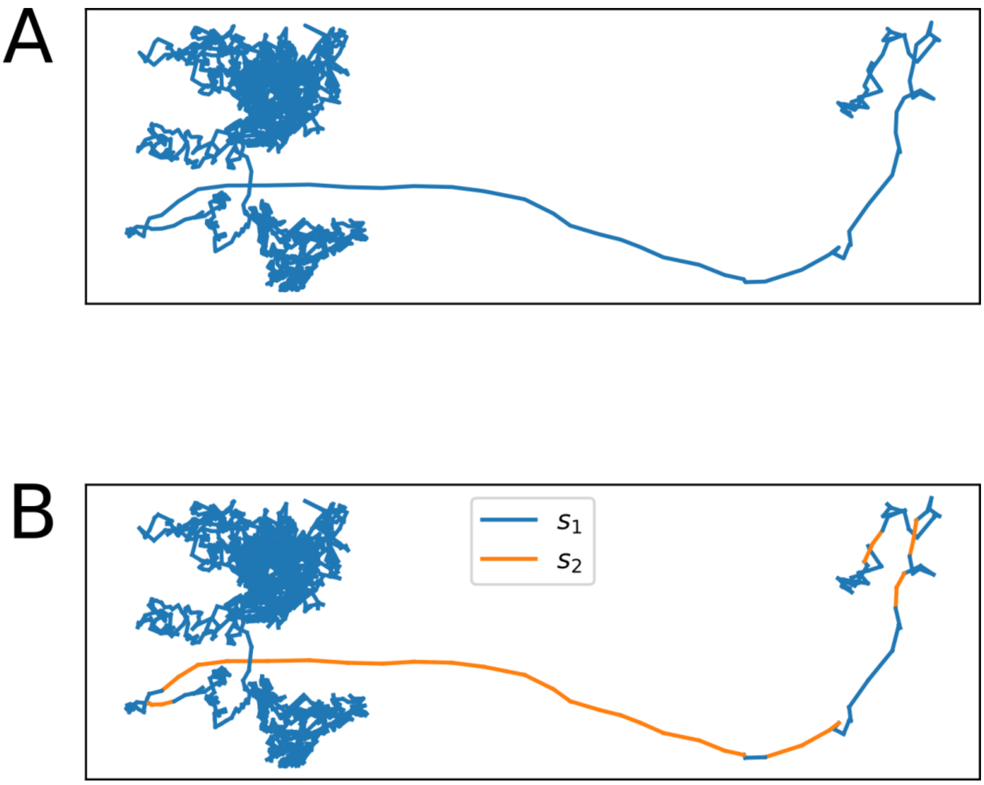

Figure S4: Example of track that was used for training the HMM. A) The original track we used for fitting parameters using Baum-Welch. B) The same track with each time step color-coded as being in s_1 or s_2 by the Viterbi algorithm.

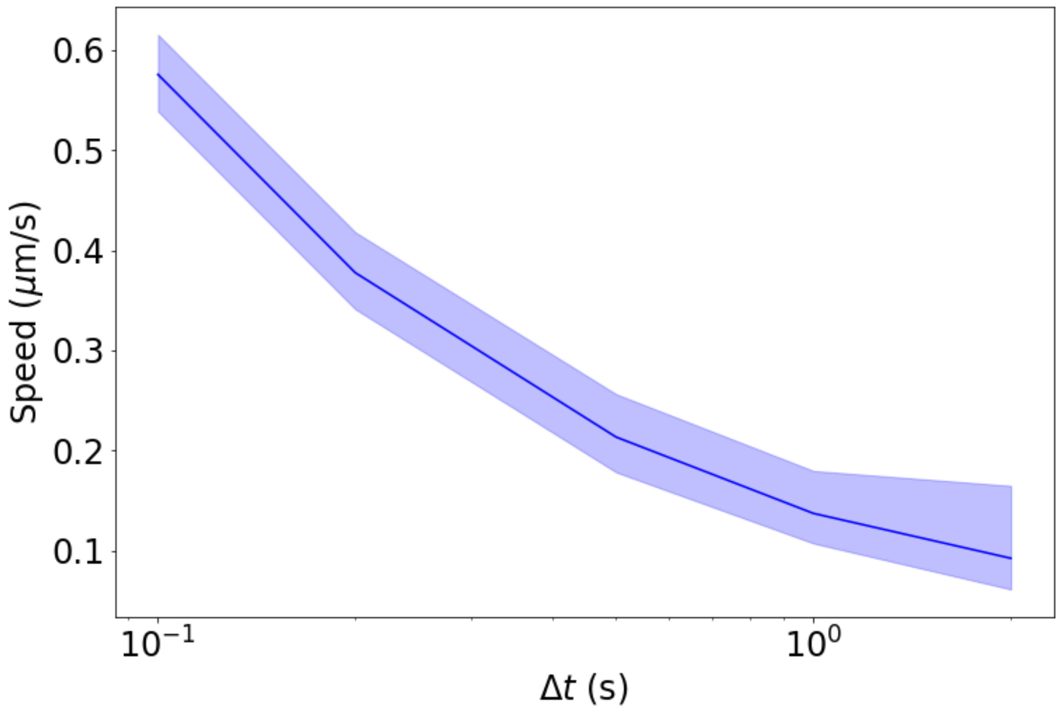

Figure S5: Estimate of instantaneous speed as a function of the frame rate. The downsampled temporal resolution is described by Δt. The shaded region indicates a 95% confidence interval of the estimated average speed. The confidence interval was calculated using bootstrapping.

Figure S6: Example video of the *norm* condition.

Figure S7: Example video of the *noc* condition.

Figure S8: Example video of the *KO PEX14* condition.

1. Monnier, N. *et al.* Inferring transient particle transport dynamics in live cells. *Nat. Methods* **12**, 838–840 (2015).
